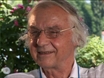# The 2008 Lindau Nobel Laureate Meeting: Robert Huber, Chemistry 1988

**DOI:** 10.3791/1128

**Published:** 2008-11-25

**Authors:** Robert Huber

## Abstract

Robert Huber and his colleagues, Johann Deisenhofer and Hartmut Michel, elucidated the three-dimensional structure of the Rhodopseudomonas viridis photosynthetic reaction center. This membrane protein complex is a basic component of photosynthesis – a process fundamental to life on Earth – and for their work, Huber and his colleagues received the 1988 Nobel Prize in Chemistry. Because structural information is central to understanding virtually any biological process, Huber likens their discovery to “switching on the light” for scientists trying to understand photosynthesis. Huber marvels at the growth of structural biology since the time he entered the field, when crystallographers worked with hand-made instruments and primitive computers, and only “a handful” of crystallographers would meet annually in the Bavarian Alps. In the “explosion” of structural biology since his early days of research, Huber looks to the rising generation of scientists to solve the remaining mysteries in the field – such as the mechanisms that underlie protein folding. A strong proponent of science mentorship, Huber delights in meeting young researchers at the annual Nobel Laureate Meetings in Lindau, Germany. He hopes that among these young scientists is an “Einstein of biology” who, he says with a twinkle in his eye, “doesn’t know it yet.” The interview was conducted by JoVE co-founder Klaus J. Korak at the Lindau Nobel Laureate Meeting 2008 in Lindau, Germany.

**Figure:**